# The effectiveness of a skin care program for the prevention of contact dermatitis in health care workers (the Healthy Hands Project): study protocol for a cluster randomized controlled trial

**DOI:** 10.1186/s13063-017-1803-0

**Published:** 2017-02-28

**Authors:** Maryam Soltanipoor, Sanja Kezic, Judith K. Sluiter, Thomas Rustemeyer

**Affiliations:** 1 Department of Dermatology, VU University Medical Centre (VUmc), De Boelelaan 1117, Amsterdam, 1081HV The Netherlands; 2 Coronel Institute of Occupational Health, Academic Medical Center (AMC), Meibergdreef 9, Amsterdam, 1105 AZ The Netherlands

## Abstract

**Background:**

Health care workers (HCW) are at high risk for developing occupational hand dermatitis (HD) due to frequent exposure to ‘wet work’. Amongst HCWs, nurses are at highest risk, with an estimated point prevalence of HD ranging between 12 and 30%. The burden of disease is high with chronicity, sick leave, risk of unemployment and impaired quality of life. Despite evidence from the medical literature on the risk factors and the importance of skin care in the prevention of HD, in practice, compliance to skin care protocols are below 30%. New preventive strategies are obviously needed.

**Methods/design:**

This is a cluster randomized controlled trial, focusing on nurses performing wet work. In total, 20 wards are recruited to include 504 participating nurses in the study at baseline. The wards will be randomized to an intervention or a control group and followed up for 18 months.

The intervention consists of the facilitation of creams being available at the wards combined with the continuous electronic monitoring of their consumption with regular feedback on skin care performance in teams of HCWs. Both the intervention and the control group receive basic education on skin protection (as ‘care as usual’). Every 6 months, participants of both groups will fill in the questionnaires regarding exposure to wet work and skin protective behavior. Furthermore, skin condition will be assessed and samples of the stratum corneum collected. The effect of the intervention will be measured by comparing the change in Hand Eczema Severity Index (HECSI score) from baseline to 12 months. The Natural Moisturizing Factor (NMF) levels, measured in the stratum corneum as an early biomarker of skin barrier damage, and the total consumption of creams per ward will be assessed as a secondary outcome.

**Discussion:**

This trial will assess the clinical effectiveness of an intervention program to prevent hand dermatitis among health care workers

**Trial registration:**

Netherlands Trial Register (NTR), identification number NTR5564. Registered on 2 November 2015.

**Electronic supplementary material:**

The online version of this article (doi:10.1186/s13063-017-1803-0) contains supplementary material, which is available to authorized users.

## Background

Health care workers (HCW) are at high risk for developing occupational hand dermatitis (HD) due to frequent exposure to ‘wet work’ [1]. Wet work, defined as unprotected exposure to humid environments/water; high frequency of hand-washing procedures or prolonged glove occlusion, is believed to cause irritant contact dermatitis in a variety of occupations [2]. Despite there being no clear scientific definition for wet work in terms of exposure frequency, duration or intensity; most study groups refer to regulatory guidelines. For example, the German guidelines, in which wet work is defined as having wet hands for more than 2 h per regular work day (per shift), hand-cleansing more than 20 times per day or the wearing of occlusive gloves for 2 h per day [3].

Amongst HCWs, nurses are particularly at high risk of HD, with an estimated point prevalence of 12–30% [4, 5]. Almost 60% of HCWs are reported to have eczema-related sick leave during the first year after notification of the disease [6]. This makes the burden of disease high for affected individuals as well as in the socioeconomic context. The total annual costs for occupational skin diseases for medical care, absenteeism and disability pensions are estimated to be €98 million in The Netherlands [7].

Recently, infection prevention policy in The Netherlands has become stricter, with emphasis on the frequent use of hand alcohol and hand washing with soap to prevent transmission of infections. This has led to an increase in exposure to irritants and a higher risk of skin barrier damage.

In The Netherlands, the Dutch Society of Occupational Medicine (NvAB) established guidelines for the prevention of occupational hand dermatitis (OHD) in 2006 [8]. The guideline stresses the importance of the maintenance of an uncompromised skin barrier for the prevention of HD and recommends regular use of skin care products such as emollients and ointments. A more recent update of contact dermatitis guidelines from The Netherlands Society of Dermatology and Venereology (NVDV) [9] consistently recommended the use of emollients to help prevent irritant contact dermatitis, referring to three studies [10–12]. The findings from one RCT showed that the use of creams decreased occupational hand dermatitis, compared to the control without any intervention [11]. In another RCT, healthy volunteers washed their hands 15 times a day, after which they used cream. Compared with the control group, skin hydration was significantly higher in the group which used creams [10]. In an experimental study by Kampf et al. [12], a test group comprising 25 subjects applied hand creams after every hand wash. The result was that the use of hydrating creams decreases the symptoms of HD including skin hydration and skin roughness. Several skin care programs have been effectively introduced in the health care setting to help prevent occupational diseases [13, 14]. The effectiveness of these programs seemed to depend on three factors: (1) the effectiveness of protective measures (i.e. skin care products), (2) the adherence to these measures (i.e. frequency of application) and (3) the effectiveness of education on preventive behavior (by raising awareness about the risk factors for HE and the importance of protective measures) [15]. Moisturizers belong to the most widely used preparations to decrease dryness and improve skin barrier function. The effects of moisturizers on the skin barrier has mainly been investigated in several experimental irritation studies [16, 17]. A number of different mechanisms behind the barrier-improving effects of various creams have been suggested, but still their mechanisms are not fully understood.

With respect to the effectiveness of education, several intervention studies have shown moderate evidence that education influences behavior, which leads to a reduction in skin symptoms [18]. Regarding the second factor, despite evidence from interventions that consider appropriate skin care effective, the adherence to these preventive measures in the workplace remains low [19].

Recently, an electronic monitoring system has been developed for the continuous registration of hand cream consumption and data recording. This system enables a detailed feedback to HCWs on the frequency with which the hand cream is used as well as when it is used. In hand hygiene studies, a similar monitoring system was earlier proven to improve compliance by 42% [20]. Monitoring and feedback are widely used as a strategy to improve professional performance and patient outcomes [21]. The effects are generally moderate and vary based on the way that the intervention is designed and delivered [22]. Feedback is suggested to be more effective to improve performance when: (1) baseline performance is low, (2) when the source is a supervisor, (3) when it is provided more than once and (4) when it is provided both verbally and in written form [22]. Group monitoring is widely recognized as being more effective than other monitoring systems that track individuals’ actions which can be seen by staff as punitive or an invasion of their privacy [23]. Providing group data to units has been shown to encourage group collaboration in a positive manner as staff work together to improve compliance, resulting in better and more sustained results.

In the present study, we aim to investigate the effectiveness of such a feedback-monitoring system combined with raising awareness in a randomized controlled trial (RCT). The intervention will comprise of the placing of hand cream dispensers on the wards, electronic monitoring of their use, and repeated feedback to the HCWs on the wards. The efficacy will be assessed by measuring skin conditions in HCWs before and after the intervention, compared with a control group.

### Objectives

We will investigate whether an intervention program, based on the provision of hand creams and regular feedback on cream consumption, can improve the skin condition in nurses engaged in wet work, when compared to ‘care as usual’.

## Methods/design

### Trial design

A two-arm, single-blinded, cluster RCT, based on hospital wards as the unit of randomization. The aim is to recruit wards and then randomly allocate them to an intervention or a control group after stratification on exposure to wet work. The follow-up is 18 months. The primary means of data collection will consist of two assessments (one clinical and the other biochemical), questionnaires and electronic consumption records.

### Trial setting

The trial will take place at a large medical academic center in The Netherlands, the VUmc (Free University Medical Center), with a total of 45 departments of which approximately 20 are clinical wards.

The investigators are responsible for the protocol, the conducting of the trial, analysis of the data and all other aspects involved.

### Recruitment of participants

The trial includes nurses engaged in wet work activities (e.g. hand washing, use of hand disinfectants, wearing gloves) on clinical wards of the VUmc. The identification of the ‘high-risk wards’ will be based on the consumption of soap and disinfectants per ward (normalized for number of HCW/ward). Wards where nurses have an increased risk of HD due to the nature of their work are defined as ‘high risk’. For recruitment, the manager responsible for a ward will receive a letter from the investigators stating the purpose of the study, a short version of the study protocol, and a brief description of the expected burden for the participant during the intervention. On the wards which are willing to participate, the researcher will give a short presentation on HD in HCWs and the preventive measures as described in the Dutch guidelines [8, 9]. During the presentation, the researcher will also inform the participants about the study and provide leaflets with study outlines. Furthermore, the HCW will be given an application form with contact details in which they can express their interest to participate in the study. The investigator will distribute the patient information letter on the wards and invite the interested participants for a visit. Nurses agree to participate by signing a Consent Form.

### Products used in the intervention group

In the electronic dispensers, placed on the intervention wards, Stokoderm® Aqua Sensitive will be used; a white, perfume- and silicone-free soft cream, with no particular pharmacological function. The main ingredients are glycerol and urea, which are known to prevent loss of water leading to dry skin.

### Inclusion criteria

- Willing to give informed written consent
- Age 18 to 65 years
- Having daily exposure to wet work activities during work
- Being employed as nurse or nutrition assistant on the participating wards

### Exclusion criteria

Being employed on more than one ward

### Description of the study procedures and intervention

**Fig. 1:**
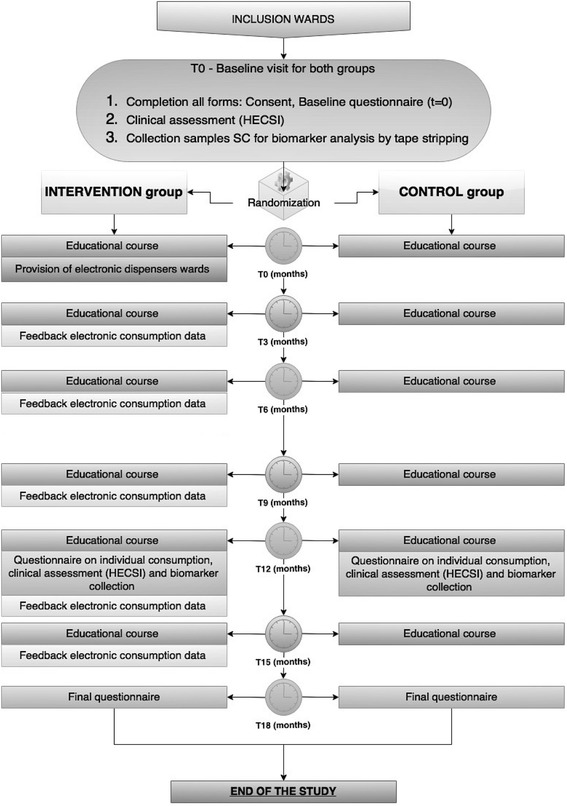
Flowchart for the Healthy Hands Project (HHP). The HHP trial will run for 18 months in total, though the primary and secondary outcomes will be assessed after 12 months, during the second and last visit

The flowchart of the study design is shown in (Fig. 1). After inclusion, the participants will fill in the baseline questionnaire and undergo baseline measurements (clinical scoring and stratum corneum (SC) collection). The baseline questionnaire includes information on participants’ characteristics (including number of working hours, and years of employment), history of atopic diseases, relevant medical history, skin condition and the risk factors/exposures (self-reported average frequency of hand washing, use of hand disinfectants, use of skin care products and gloves during a shift). Filling in the questionnaires will take approximately 3–5 min per participant.

Both the intervention and the control group will receive basic education on skin protection (as ‘care as usual’). These educational courses will be given on each ward every 3 months from baseline to the end of study. Our group education program (as described in Table 1) will comprise basic knowledge about the skin, the development of eczema and recommendation for skin protection, and care as proposed by the NVAB guidelines for contact dermatitis [8] (Table 2).

**Table 1:** Educational program

Methods	ᅟ
-Educational courses every 3 months	ᅟ
-Booklet on preventive measures	ᅟ
Program topics	ᅟ
-What is hand eczema and what are the symptoms?	ᅟ
-Risk factors of hand eczema	ᅟ
-Consequences of hand eczema	ᅟ
-Preventive measures	ᅟ

**Table 2:** Main recommendations for the prevention of hand dermatitis (NVAB guideline, 2006) [8]

1. Use disinfectants instead of water and soap to disinfect the hands, when hands are not visibly dirty	ᅟ
2. Wear gloves when performing wet work	ᅟ
3. Wear cotton under-gloves when you wear gloves for longer than 10 min	ᅟ
4. Use a moisturizer on a daily basis to nurse the skin	ᅟ
5. Creams should be applied over the whole hand, including the webs, fingertips and dorsal aspects	ᅟ

At baseline and 12 months after baseline, the participants in both groups will undergo clinical measurements (Hand Eczema Severity Index (HECSI) scoring), SC samples will be collected for Natural Moisturizing Factor (NMF) analysis and the participants will fill in the questionnaire.

Participants in the control group (just as in the intervention group) with severe eczema requiring medical treatment will be advised to consult the occupational physician or dermatologist.

Additional procedure in the intervention group:

After randomization into the intervention group, the hand cream (Stokoderma® Aqua Sensitive) will be provided in electronic dispensers (with monitoring system) on the wards at places which are easily accessible. At least five dispensers per ward will be located at sinks, at the entrance and exit of each ward and other relevant places such as in coffee rooms and toilets. The provided hand cream is commercially available and is widely used in the health care sector.

Each dispenser records continuously each application event, providing information on the timing and frequency of use of hand creams during the working shift. The system provides robust and easy to interpret web-based reports on cream use per dispenser. Data on use pattern (frequency, total consumption, moments of use) and trends will enable a structured feedback on hand cream use to the nurses and management to motivate and improve compliance. Feedback sessions on hand cream use will be done every 6 months after baseline during regular meetings of the nursing staff and performed by the head nurse.

### Outcome measures

#### Primary outcome

The change in disease severity as assessed by the Hand Eczema Severity Index (HECSI) score.

This validated scoring system, the HECSI [24], grades the intensity of erythema, induration, papules, vesicles, fissures, scaling and oedema for five areas of each hand (fingertips, fingers, palms, back of hands, wrists) on a scale from 0 (not present) to 3 (severe). The extent of affected skin in each area is graded from 0 to 4. The total index score with a range from 0 to 360 is then found by multiplying the intensity with the extent [24]. The HECSI will be assessed at baseline and after 12 months in both the intervention and the control group.

### Secondary outcomes

The change in levels of NMF in the skin as a marker of early signs of barrier damage.

NMFs are mainly composed of the breakdown products of epidermal protein filaggrin. NMFs play an important role for the skin barrier function as they contribute to skin hydration, the maintenance of the acidic pH of the skin, and the epidermal inflammatory response [25]. Recently, it has been shown that various skin irritants significantly reduce the levels of NMF [26]. One study showed that during cleansing (and more than 10-min water contact) large quantities of NMF can leach from the skin surface, leading to dry skin and, by repetitive exposures to skin barrier damage, to inflammation [27]. Therefore, the NMF levels might reflect early damage of the skin barrier.

The NMF levels will be determined in the uppermost layers of the skin, the stratum corneum (SC). The SC samples will be harvested by using adhesive tape strips, a method which is extensively used in experimental studies [28]. Briefly, round adhesive tape discs (3 × 8 cm^2^, D-Squame; CuDerm, Dallas, TX, USA) will be attached to the skin of the right hand. Each tape is pressed onto the volar aspect of the hand for 10 s with standardized force, using a disc pressure applicator (CuDerm, Dallas, TX, USA). The first four successive strips from the same skin site will be discarded and for the NMF analysis the fifth to seventh tape strips will be collected. The tape strips are gently removed with tweezers and stored in a closed vial at −20 °C until analysis. For the analysis of NMF in tape strips, NMF constituents will be extracted from the tape strips using 40% ammonia and subsequently analysed by HPLC-UV [29]. To compensate for variable amount of SC harvested by a tape, the protein amount will be determined on each tape by measuring optical density (SquameScan, CuDerm, Dallas, TX, USA). NMF concentration on the tape will be normalized by the protein amount.

### Process outcomes

Individual consumption of hand creams (application frequency/per shift) will be assessed by questionnaires at baseline and every 6 months from baseline.

Total consumption of creams in the intervention group will be measured by real-time monitoring per ward as a secondary outcome and compared over time.

### Skin exposure to irritants

Individual exposure will be assessed by questionnaires (estimated frequency of soap and alcohol use per shift), completed every 6 months. Furthermore, exposure per ward will be estimated from data on the purchase of soap and disinfectants.

### Sample size

This trial is planned to include 544 individuals. The sample size is based on a previous study, in which the Osnabruck Hand Eczema Severity Index score after 12 months’ follow-up in the control group was 0.1 points with a standard deviation of 1.2. A difference in the Osnabruck Hand Eczema Severity Index score of 0.4 points is clinically significant [30]. Using a two-sided *t* test with a significance level of 0.05, we calculated that a study with 17 clusters per treatment group with 16 individuals per cluster would have 81% power to detect a difference of 0.4 in group means when the standard deviation is 1.2 and the intracluster correlation is 0.05.

### Randomization

The method of fixed-block randomization will be used to carry out randomization with block sizes of 2. The blocks (one high-exposure block and one low-exposure block) will be stratified by exposure to wet work, estimated from the purchase of soaps per ward. Two blocks will be randomized at a time to reduce bias and achieve balance in the allocation of participants to the intervention or control arm. Randomization will be performed prior to baseline collection.

The investigator, assessing the clinical outcomes, will be blinded with respect to treatment allocation until after analysis.

### Statistical methods and data analysis

We will use state-of-the-art methods to deal with missing data and statistical methods to analyse our data and publish a full statistical analysis plan before the researchers are unblinded. When reporting the results of this study, we will adhere to the Consolidated Standards of Reporting Trials (CONSORT) Statement [31] and its extensions on the reporting of patient-reported outcomes in randomized trials [32] and on cluster randomized trials [33].

### Blinding

The investigator, assessing the clinical outcome, will be blinded with respect to treatment allocation. It is not possible to blind the participants.

### Participants’ withdrawal

Subjects are free to leave the study at any time for any reason if they wish to do so, without any consequences. The investigator can decide to withdraw a subject from the study for urgent medical reasons.

### Finance and insurance

The trial is (partly) financed by an unrestricted grant from the company DEB, which covers all expenses related to the study. The participants in the study are covered by their work insurance.

## Discussion

The overall purpose of the Healthy Hands Project is to change the behavior of HCWs towards hand care and make skin care part of the work culture. We look at HD among nurses, who are known to have an increased risk of occupational hand eczema due to wet work. We focus on the facilitation of cream availability combined with the continuous monitoring of its consumption with regular feedback on skin care performance in HCWs, aimed at improving their preventive behavior. In addition, this trial will provide information on the actual degree of exposure to wet work in HCWs in The Netherlands, which is important for focused prevention.

The intervention is general, straightforward and, therefore, easy to implement in health care institutions. The results of this study will help to gain insight into the effectiveness of our intervention program on skin condition and, secondarily, on the consumption of hand creams. We will provide relevant data on the current use of moisturizers in practice in health care work environments and the efficacy of an intervention which is relatively easy to implement.

Currently, a multicenter intervention RCT, which investigates a range of interventions aimed at the reduction of hand dermatitis among nurses, is running in the UK (the SCIN trial) [34]. In contrast to our study, the SCIN trial focuses on behavioral change to improve hand care, based on the theory of planned behavior and implementation intentions. The impact of the interventions will be assessed via questionnaires and standardized photographs of hands/wrists, and the primary outcome parameter will be the change in point prevalence of visible hand dermatitis from baseline to 12 months after the intervention. In the present study, the changes in the skin condition between control and intervention groups are used as the primary outcome. The skin condition will be assessed by clinical scoring (HECSI score) performed by a trained physician. In addition, we include as a secondary outcome the NMF levels, which might be a more sensitive parameter of the skin damage than clinical scoring.

With an RCT design, the risks of selection bias will be reduced, and random and design errors limited [30]. The risk of bias will be further reduced by conducting a central randomization stratified for exposure to soaps as an important prognostic factor. We blinded the outcome assessors to minimize the risk of detection bias. In order to limit contamination, the control group will not receive information about the existence of an intervention group or the purpose of the study [35]. It must be acknowledged, however, that a risk of contamination bias cannot entirely be avoided because the participants work in the same medical center and could find out about the intervention group.

Strong points*:* real-time monitoring of consumption of creams, objective assessment of the skin condition, facilitation of creams, feedback on performance, and determining NMF levels to detect early signs of skin barrier damage.

Drawbacks: self-reported hand cream use to assess individual use of skin care in both the control group and the intervention group. The objective data on cream use in the intervention group are ward-based. Exposure estimates are ward-based and self-reported at the individual level.

### Trial status

Recruitment into this trial started in June 2016 and is taking place in the VU Medical Center in Amsterdam. Patient recruitment has not been completed at the time of submission.

## Additional file

Additional file 1: Completed SPIRIT Checklist. (DOC 121 kb)

## Abbreviations

(O)HD: (Occupational) hand dermatitis
AMC: Academic Medical Center
HCW: Health care worker
HE: Hand eczema
HECSI: Hand Eczema Severity Index
HHP: Healthy Hands Project
METC: Medical Research Ethics Committee (MREC); in Dutch: medisch ethische toetsing commissie (METC)
NMF: Natural Moisturizing Factor
NvAB: Netherlands Society for Occupational Health; in Dutch: Nederlandse Vereniging voor Arbeid en Bedrijfsgeneeskunde
NVDV: Netherlands Society of Dermatology and Venereology; in Dutch: Nederlandse Vereniging voor Dermatologie en Venereologie
RCT: Randomized controlled trial
SC: Stratum corneum
